# Evaluation of Nuclear Morphometry and Ki-67 Index in Clear Cell Renal Cell Carcinomas: a Five-Year Study

**Published:** 2017-04-01

**Authors:** C S Sheela devi, S Suchitha, R S Veerendrasagar

**Affiliations:** 1 *Dept. of Pathology, JSS Medical College and Hospital, Mysore, Karnataka, India*

**Keywords:** Clear Cell Renal Cell Carcinoma, Fuhrman Grade, Nuclear Morphometry, Ki-67

## Abstract

**Background and objective:**

Clear Cell Renal Cell Carcinoma (CCRCC) is the most common adult renal neoplasm. Staging and grading of RCC are important predictors of survival. Fuhrman nuclear grading is widely used for CCRCC, the subjective nature of which has prompted more objective methods to evaluate nuclear features. Furthermore, Ki-67, a reliable marker of cellular proliferation may provide another variable for assessment of the biological behavior of RCC. The aim of this research was to study nuclear morphometry and Fuhrman nuclear grading of clear cell RCC, and to assess their relationship with the Ki-67 index.

**Methods:**

Hematoxylin and eosin slides of forty cases of CCRCC were retrieved and studied for pathologic variables, including Fuhrman nuclear grade, pathological tumor and node stage. Nuclear morphometric analysis was performed using computer-assisted image analysis. The relationship between Fuhrman nuclear grading, pathologic stage, tumor size, nuclear morphometry and proliferative index were analyzed.

**Results:**

According to Fuhrman grading, four (10%) cases were grade I, 23 (57.5%) were grade II, 12 (30%) were grade III, and one (2.5%) was grade IV. Moderate to high correlation was seen between Fuhrman nuclear grade and mean nuclear area, perimeter, diameter, length, nuclear roundness factor and Ki -67, with a P value of < 0.05.

**Conclusion:**

The CCRCC is an extremely heterogenous disease and clinical outcome is unpredictable despite several validated prognostic factors. The widely used Fuhrman nuclear grading is subjective, while nuclear morphometry, using computer assisted image analysis, can ensure more objective assessment. The Ki-67 index could provide reliable information and compliment the other prognostic parameters.

## Introduction

Renal Cell Carcinoma (RCC) accounts for about 3% of adult malignancies and 90% to 95% of neoplasms arising from the kidney (1,2). It is a clinicopathologically heterogeneous disease with several histologically distinctive subtypes, which differ in morphology, molecular genetics and pathogenesis (3,4). Clear Cell RCC (CCRCC) is the most common subtype, accounting for 70% of all renal neoplasms (2). The incidence of RCC is increasing and it is highly unpredictable with a tendency for recurrence. This disease could be the cause of death many years after initial treatment (3).

Staging and nuclear grading of RCC are considered as important predictors of survival. Several systems have been proposed for the grading of renal cell carcinoma (5). Fuhrman nuclear grading system, based on nuclear size, nuclear shape and prominence of nucleoli is widely used for RCC. However, it is limited by its subjective nature and low reproducibility, which has necessitated quantitative morphometric approaches to evaluate nuclear features (3,6). Nuclear morphometry, using computer-assisted image analysis, is the most commonly used system for this purpose. It can overcome intraobserver and interobserver variations and in conjunction with histopathological grading, may ensure more objective assessment of RCCs (7,8).

 Cellular proliferation rate may provide another predictive variable for the biological aggression of RCC, and this could be evaluated by the study of Ki-67 (kiel67) antigen. It is an easy and reliable marker that could be applied on formalin-fixed tissue for better assessment of the biological behavior of RCC and prediction of the patient’s outcome (9). 

 The objective of this study was to determine the correlation between Fuhrman nuclear grading with nuclear morphometry using computer assisted image analysis and Ki-67 proliferation index in clear cell renal cell carcinoma.

## Material and Methods:

Forty histopathologically diagnosed clear cell renal cell carcinoma in nephrectomy specimens were included in the study from 2011 to 2015. Consent was obtained from the institutional ethical committee.

The paraffin blocks were retrieved and stained with Hematoxylin and Eosin (H&E). The pathologic variables, including tumor size, Fuhrman nuclear grade and pathological staging, according to Tumor, Node and Metastasis (TNM) were recorded.

Nuclear morphometric analysis was performed on H&E stained histologic sections, using Olympus BX-41 research microscope with Jenoptix (Germany) progress charge-coupled device (CCD) camera and progress capture pro-imaging software. The digital images were captured with 1X C mount CCD adapter. After transferring microscopic images to the computer, morphometric parameters were measured by the image analysis program. About 100 nuclei from each case with sharply demarcated contours were included for morphometric analysis in the highest grade area of the tumor. The following nuclear morphometric parameters were recorded: Mean Nuclear Area (MNA), Mean Nuclear Perimeter (MNP), Mean Nuclear Length (MNL), and Mean Nuclear Diameter (MND). All measurements were made under 400X magnification and expressed in microns. Two parameters were calculated including, Mean Nuclear Roundness Factor (MNRF), which is equal to perimeter^2^/4𝜋 area, and Mean Nuclear Form ellipse (MNFe) which is the longest diameter/ the shortest diameter (6).

Immunohistochemistry was performed on 4-µm thick sections on poly-l-lysine coated slides. Antigen retrieval was done using citrate buffer at pH 9.2. Monoclonal antibody Ki-67 (Novocastra, code no Ki-67-MM1-R7-C) was used for Ki-67 antigen detection by standard streptavidin–biotin technique using anovostain universal detection kit (Novocastra, code no.RTU-Ki-67-MM1). Sections from a reactive lymph node were taken as positive control, whereas sections treated with tris-buffer solution instead of the primary antibody, were used as the negative control. Brown granular nuclear reactivity was considered as positive. An area with maximum proliferation was chosen to evaluate the Labeling Index (LI). Labeling Index is expressed as percentage of positively stained nuclei per 100 epithelial cells after counting at least 1000 cells in each case under 400 X magnification (10).

The Fuhrman nuclear grading was independently recorded by two observers. Kappa statistics were used to evaluate the concordance between the two observers with regards to Fuhrman nuclear grading (fair agreement, κ = 0.00 to 0.20; moderate agreement, κ = 0.21 to 0.45; substantial agreement, κ = 0.46 to 0.75; near perfect agreement, κ = 0.76 to 0.99; perfect agreement, κ = 1.00) (7). Finally, both pathologists arrived to a consensus, which was later subjected to nuclear morphometry. The chi square test was used for comparing the results of the two independent observers with the final results after consensus.

The statistical analysis was performed using descriptive statistics, independent sample *t* test analysis, correlation and one-way Analysis of Variance (ANOVA). The SPSS software (version 16.0) and Minitab (version 11.0) were used for data analysis.

The relationship between Fuhrman nuclear grading, pathologic stage, tumor size, nuclear morphometric results and proliferative index were determined by the Pearson correlation coefficient. P values of<0.05 were considered statistically significant.

The age of the patients ranged from 24 to 80 years with a mean of 58.05 years. The majority of the patients were aged between 61 and 70 years. The male:female ratio was 3:1. Based on the size of the tumor in centimeters (cm), tumors were categorized to three groups, group one: 1 to 7 cm (n=21), group two: 7.1 to14 cm (n=17) and group three: >14cm (n=2).

**Nuclear morphometric parameters**

In the present study, Fuhrman nuclear grading was initially performed independently by two observers (I and II) with moderate agreement, and a kappa value of 0.45. Final grading (III) was then subsequently recorded with consensus between the two observers and there was substantial agreement with a kappa value of 0.67 (Table 1).

**Table 1:** Fuhrman Nuclear Grading of Clear Cell Renal Cell Carcinoma by Two Observers and Final Grading With Consensus

Observers	Grade 1	Grade 2	Grade 3	Grade 4	Chi	P
I	4	18	16	2	2.49	0.879 *df=6
II	5	19	13	3		
III	4	23	12	1		

* df – Degrees of freedom

The nuclear grade was assigned to the least differentiated area of the tumor, in accordance to the criteria of Fuhrman et al. (7). The majority were grade 2 (57.5%) followed by grade 3 (30%), grade 1 (10%), and grade 4 (2.5%). Fourteen (35%) patients were in stage 3, followed by 13 (32.5%) each in stage 1 and 2.

**Fig 1:**
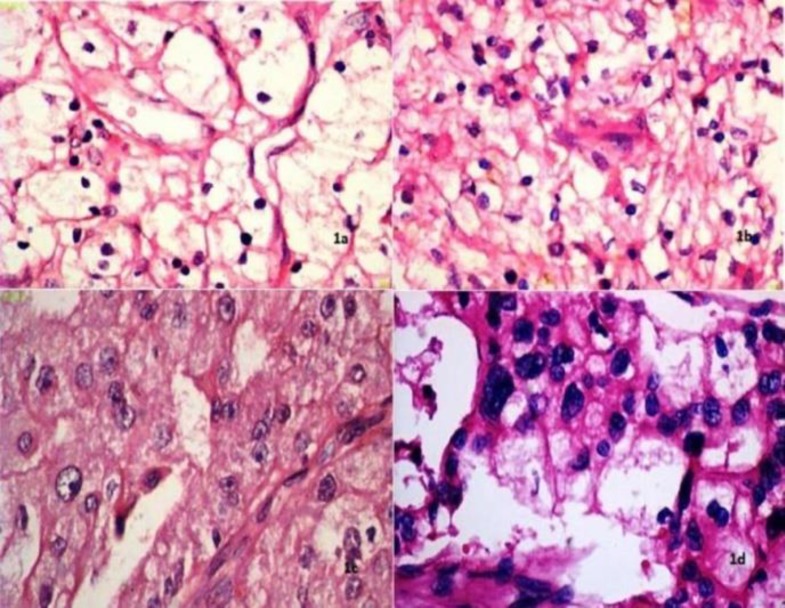
Fuhrman nuclear grading in clear cell RCC (H&E, x400)

**Fig 2:**
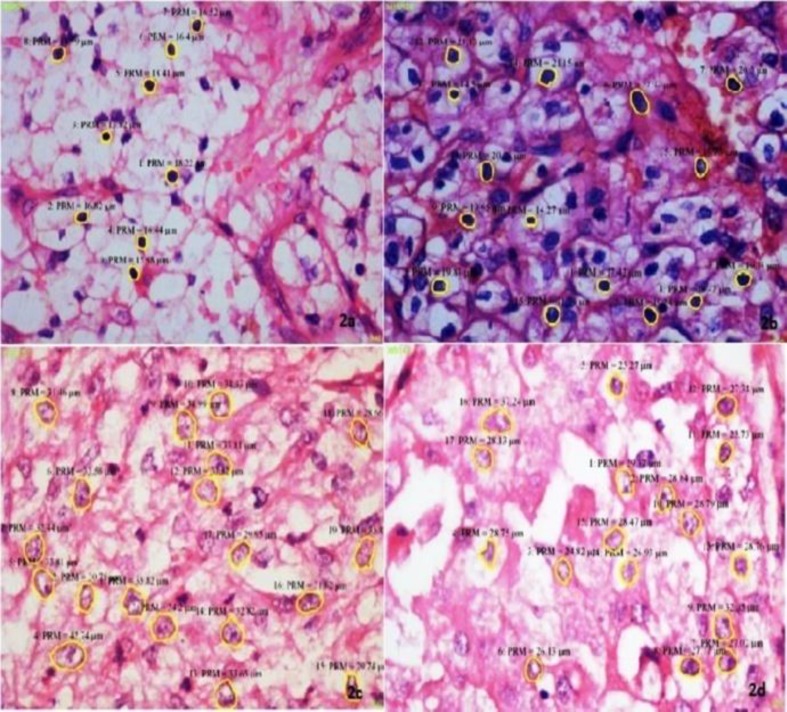
Nuclear morphometry in clear cell RCC (H&E, x400)

**Fig 3:**
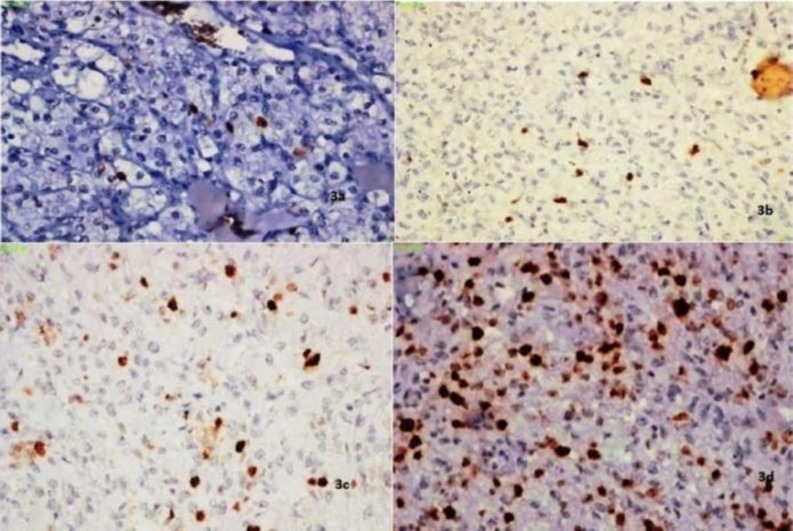
IHC of Ki-67 in clear cell RCC (x200)

The mean nuclear area, mean nuclear diameter and mean nuclear length, had moderate correlation with the Fuhrman grade, and the mean nuclear perimeter had good correlation with the Fuhrman grade. The mean nuclear roundness factor negatively correlated with the Fuhrman grade. (Fig 1 & 2) Immunohistochemical (IHC) expression of Ki-67 (%) (Fig 3)

a) Clear cell RCC showing Fuhrman nuclear grade 1 (H&E, x 400)

b) Clear cell RCC showing Fuhrman nuclear grade 2 (H&E, x 400)

c) Clear cell RCC showing Fuhrman nuclear grade 3 (H&E, x 400)

d) Clear cell RCC showing Fuhrman nuclear grade 4 (H&E, x 400)

a) Nuclear Morphometry in Fuhrman grade 1 of Clear cell RCC (H&E, x 400)

b) Nuclear Morphometry in Fuhrman grade 2 of Clear cell RCC (H&E, x 400)

c) Nuclear Morphometry in Fuhrman grade 3 of Clear cell RCC (H&E, x 400)

d) Nuclear Morphometry in Fuhrman grade 4 of Clear cell RCC (H&E, x 400)

The Ki-67 expression ranged from 12.5% to 55% and the mean expression had moderate correlation with Fuhrman grade; Pearson correlation coefficient of 0.674 and a P value of <0.001. However, there was poor correlation with tumor size.

With regards to comparison of tumor size, pathological tumor staging, nuclear morphometry and Ki-67 proliferation, there was an increase in Mean Nuclear Area (MNA), Mean Nuclear Perimeter (MNP), Mean Nuclear Length (MNL), Mean Nuclear Diameter (MND), and Ki-67 proliferation, proportional to higher stages and increase in tumor size. However, only MNFe was statistically significant with a P value of 0.004, in relation to pT stage (Table 2). 

a) IHC of Ki-67 (%) in Fuhrman grade1 of Clear cell RCC(x200)

b) IHC of Ki-67 (%) in Fuhrman grade2 of Clear cell RCC(x200)

c) IHC of Ki-67 (%) in Fuhrman grade3 of Clear cell RCC(x200)

d) IHC of Ki-67 (%) in Fuhrman grade 4 of Clear cell RCC(x200)

## Discussion

The CCRCC is a very heterogenous disease, and surgery remains the only curative therapy despite the introduction of a number of new promising treatment options (11,12).The precise identification of prognostic factors is therefore an essential step in the evaluation of CCRCC (13).The most important parameters regarding the potential clinical and biological outcome of the tumor are tumor stage and grade (9). The most commonly used and widely accepted method is Fuhrman nuclear grading system, which depends on the outlines of cell nuclei, nuclear size, presence or absence of nucleoli (9,13). It is recognized as being highly subjective with low to moderate interobserver agreement (13). Many issues were encountered with the Fuhrman nuclear grading system with regards to reproducibility, inter-observer variability and objectivity, and major criticism against this grading is the difficulty in differentiating intermediate grades, which possibly contributes to lack of uniformity in the use of nuclear grading (3,13).

**Table 2:** Correlation Between Tumor Size, Fuhrman Grade, Pathological Tumor Stage, Ki-67 and Nuclear Morphometry

Fuhrman Grade	MNA (µm^2^)	MNP (µm)	MND (µm)	MNL (µm)	MNRF (µm)	MNFe (µm)	Mean Ki-67 %
1 (n=4,10%)	54.51	28.23	8.88	8.32	1.17	1.96	12.50
2 (n=23,57.5%)	59.28	29.11	8.89	8.93	1.14	1.95	15.04
3 (n=12,30%)	74.93	33.85	10.32	10.37	1.10	1.84	31.17
4 (n=1,2.5%)	117.33	39.20	11.88	11.81	1.04	1.81	55.00
p value	<0.000	<0.000	<0.000	<0.000	0.003	0.146	<0.000
Tumor Size (cm)							
Group 1 (1-7 cm) (N=21, 52.5%)	59.36	30.09	9.21	9.25	1.14	1.94	17.80
Group 2 (7.1 -14 cm) (n=17, 42.5%)	68.77	30.94	9.50	9.41	1.12	1.89	21.64
Group3 (>14.1cm)(n=2,5%)	91.16	35.05	10.44	10.43	1.13	1.85	41.50
p value	0.020	0.114	0.223	0.353	0.580	0.520	0.038
pT staging							
Stage 1(n=13,32.5%)	60.70	29.41	8.98	9.04	1.14	1.98	14.84
Stage 2 (n=13,32.5%)	68.37	30.72	9.46	9.42	1.11	1.81	20.69
Stage 3 (n=14,35%)	65.72	31.87	9.72	9.65	1.13	1.94	25.92
p value	0.538	0.153	0.161	0.349	0.247	0.004	0.81

Mean Nuclear Area (MNA), Mean Nuclear Perimeter (MNP), Mean Nuclear Length (MNL), Mean Nuclear Diameter (MND), Mean Nuclear Roundness Factor (MNRF) and Mean Nuclear Form ellipse (MNFe)

In the present study, Fuhrman nuclear grading was done by two observers and there was moderate agreement between them with kappa value of 0.45. A multi-centered study to assess inter-observer agreement between three pathologists, using the Fuhrman grading system, observed low-to-moderate agreement with kappa value of 0.22. The moderate level of inter observer agreement can be explained by the element of subjectivity in estimating the nuclear size, and the heterogeneous nature of the tumor meant that it is composed of cells of different grades (13). Al-Ayanthi et al. (14) found moderate inter observer agreement with a mean k value of 0.29. 

When grading was done with consensus between the two observers in the present study, there was substantial agreement with kappa value of 0.67 and majorityof the cases were Fuhrman grade 2, followed by grade 3, grade 1 and grade 4 (1,6). Lang H et al. (13) obtained the best concordance by collapsing to a two-tiered system of low grade (grade 1 to 2) and high grade (grade 3 to 4) without a significant loss of information regarding survival. It has been proposed to reduce the grades in the Fuhrman system for better outcome stratification (8,15).

The grading system based on standardized and reproducible criteria that reflect the heterogeneity of nuclear and nucleolar features are recommended by the Union Internationale Contre le Cancer (UICC) and **American Joint Committee on Cancer **(AJCC). Nuclear morphometry, which describes the size or shape of the nuclei, is the most commonly used system for this purpose (8). Nuclear morphometryis achieved with computer imaging systems that provide a useful and reproducible method (3,15).

Nuclear morphometric parameters have been compared with conventional grading systems for malignancies of various organs, and several authors have tried to introduce objective measures for nuclear grading in CCRCC.

In the present study, nuclear morphometric parameters including Mean Nuclear Area (MNA), Mean Nuclear Perimeter (MNP), Mean Nuclear Length (MNL), Mean Nuclear Diameter (MND),Mean Nuclear Roundness Factor (MNRF) and Mean Nuclear Form ellipse (MNFe) were analysed.

The MNA and MND moderately correlated with Fuhrman grade, which is concordant with various studies (3,6,9).

Delahuntet al. (16) undertook a study to determine the relationship of the three morphologic components of the Fuhrman grading system and also to determine if they were correlated with outcome for clear cell renal cell carcinoma. On multivariate analysis, worst nucleolar grade retained a significant association with survival when modeled with nuclear area. They showed that the association of worst nucleolar grade with outcome was independent of nuclear area, whereas it was a dependent variable when tested against other parameters of nuclear size (16). A significant correlation was noted between mean major nuclear diameter and certain clincopathologic parameters, including tumors with sarcomatoid differentiation, perinephric fat invasion, renal capsule invasion and Fuhrman nuclear grade (8).

In the present study, mean nuclear perimeter had good correlation with Fuhrman grade and mean nuclear length had moderate correlation with Fuhrman grade (3,6). 

Mean Nuclear Roundness Factor and MNFe are shape descriptors and yield a minimal value of 1 for a perfect circle and increase as the shape of a contour deviates from circularity. In the present study, MNRF and MNFE negatively correlated with Fuhrman grade, which showed a decrease as the Fuhrman grade increased. However, MNFe was not statistically significant. These findings are comparable with other studies (6).Descriptors of nuclear shape have yielded variable results as predictors of outcome. Carducci et al. (17) found them to be useful for the identification of patients with adverse outcome.Ozer et al. (3) found a relationship between higher MNFE and sarcomatoid histology, while other authors, including Ruiz Cerda et al.(18) found it to be less reproducible and attributed this to the inaccuracy due to the handdrawing of the nuclear contour with the cursor; this could be the possible explanation for the statistically insignificant MNFE that was noted in the present study (3,18). 

An isolated assessment of a quantitative feature may not suffice to describe nuclear abnormalities and the combination of more features may be required to enable an accurate prediction of prognosis. 

Delahunt et al. (16) indicated that worst nucleolar grading alone was a valid grading parameter for CCRCC. Morphometry of nucleoli, however, was not done in this study.

Age of the patient is an independent prognostic factor and studies have shown an increase in the incidence of tumor with age. However, the morphology and clinical behavior with respect to age of the patient is still equivocal (19,20). There was no significant relationship between patient gender and other variables (19).

In the present study, 13 cases (32.5%) were in pathological stage 1 and stage 2, and 14 cases (35%) in stage 3 (Table 2). When it was compared with nuclear morphometry, there was an increase in the MNA, MNP, MNL and MND at higher stages. Only MNFe was statistically significant. Bektas et al. (8) observed a moderate correlation between MNA, MNL, MNB, MNP, MNRF and pathological stage. The mean nuclear area and mean nuclear diameter increased significantly with increasing stage (3,9).

There was an increase in MNA, MNP, MNL and MND with an increase in tumor size. However, only MNA correlated,though poorly, with tumor size (P value of 0.020) (6).

The heterogeneity of RCC within the same tumor stage and grade has necessitated the need for more specific prognostic markers related to molecular mechanisms of RCC. Additional markers, that are still under investigation include cellular proliferation, apoptosis and angiogenesis (21).The rate of cell proliferation is thought to have a major influence on tumor behavior, and Ki-67 immunostaining is a clinically applicable, rapid, reproducible method, which serves as a good marker for proliferative activity in cell nuclei (15,20,22).

The immunohistochemical expression of Ki-67 ranged from 12.5% to 55%. It moderately correlated with Fuhrman grade and poorly correlated with tumor size with a P value of <0.001 and 0.034, respectively, which was in concordance with other studies (3,9,10,12, 20,22, 23,24,). There was a proportional increase in the expression of Ki-67 with higher stage. However, the P value was 0.81, which was not statistically significant. 

The analysis of post treatment recurrence, overall survival and disease-free survival was not possible as the patients were lost to follow up. 

## Conclusion

Clear Cell Renal Cell Carcinoma is a heterogeneous disease. The stage and Fuhrman nuclear grade are considered as the most important predictors. In this study, Fuhrman grading was done by two observers, which showed moderate agreement, and was slightly improved on revising the grading with consensus. Nuclear morphometry using computer-assisted image analysis was used to ensure more objective assessment of histological grading. Though it is a well-established fact that nuclear morphometry is objective and reproducible, it is essential to incorporate it in routine reporting.

The Ki-67 labelling index may provide reliable information and compliment the other prognostic parameters in clear cell RCC. In the present study, there was moderate correlation between Fuhrman grade and Ki-67. However, the correlation was poor between tumor size, pathological stage and Ki-67, though there was an increase in the expression of Ki-67 with an increase in tumor size and higher pathological stage. 

Despite understanding conventional clinical and pathological factors, the biologic behavior of RCC is unpredictable. Therefore, there is a need for evaluation of multiple parameters in combination with biological markers that predict tumor aggressiveness.
